# Metavalent Bonding in Layered Phase‐Change Memory Materials

**DOI:** 10.1002/advs.202300901

**Published:** 2023-03-30

**Authors:** Wei Zhang, Hangming Zhang, Suyang Sun, Xiaozhe Wang, Zhewen Lu, Xudong Wang, Jiang‐Jing Wang, Chunlin Jia, Carl‐Friedrich Schön, Riccardo Mazzarello, En Ma, Matthias Wuttig

**Affiliations:** ^1^ Center for Alloy Innovation and Design (CAID) State Key Laboratory for Mechanical Behavior of Materials Xi'an Jiaotong University Xi'an 710049 China; ^2^ School of Microelectronics State Key Laboratory for Mechanical Behavior of Materials Xi'an Jiaotong University Xi'an 710049 China; ^3^ Institute of Physics IA JARA‐FIT RWTH Aachen University 52074 Aachen Germany; ^4^ Department of Physics Sapienza University of Rome Rome 00185 Italy; ^5^ Peter Grünberg Institute (PGI 10) Forschungszentrum Jülich GmbH 52425 Jülich Germany

**Keywords:** atomic imaging, metavalent bonding, optical properties, phase‐change materials, van der Waals

## Abstract

Metavalent bonding (MVB) is characterized by the competition between electron delocalization as in metallic bonding and electron localization as in covalent or ionic bonding, serving as an essential ingredient in phase‐change materials for advanced memory applications. The crystalline phase‐change materials exhibits MVB, which stems from the highly aligned *p* orbitals and results in large dielectric constants. Breaking the alignment of these chemical bonds leads to a drastic reduction in dielectric constants. In this work, it is clarified how MVB develops across the so‐called van der Waals‐like gaps in layered Sb_2_Te_3_ and Ge–Sb–Te alloys, where coupling of *p* orbitals is significantly reduced. A type of extended defect involving such gaps in thin films of trigonal Sb_2_Te_3_ is identified by atomic imaging experiments and ab initio simulations. It is shown that this defect has an impact on the structural and optical properties, which is consistent with the presence of non‐negligible electron sharing in the gaps. Furthermore, the degree of MVB across the gaps is tailored by applying uniaxial strain, which results in a large variation of dielectric function and reflectivity in the trigonal phase. At last, design strategies are provided for applications utilizing the trigonal phase.

## Graphical abstract

This work clarifies how metavalent bonding develops across the van der Waals‐like gaps in trigonal Sb_2_Te_3_ and Ge‐Sb–Te alloys, where the coupling of *p* orbitals is significantly reduced. The non‐negligible electron sharing in the gaps can be tailored by applying uniaxial strain, resulting in a large variation of dielectric function and optical reflectivity in trigonal Sb_2_Te_3_.

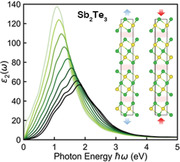

## 1 Introduction

Chalcogenide phase‐change memory materials (PCMs),^[^ ^1^, ^2^, ^3^, ^4^, ^5^, ^6^, ^7^, ^8^, ^9^, ^10^, ^11^, ^12^, ^13^, ^14^, ^15^, ^16^, ^17^, ^18^, ^19^, ^20^ ^]^ in particular, Ge–Sb–Te (GST) alloys along the GeTe–Sb_2_Te_3_ pseudo‐binary line,^[^ ^1^ ^]^ have enabled a wide range of electronic and photonic applications. The GST‐based 3D Xpoint memory is commercially available and serves as a critical component to bridge the performance gap between memory and storage units for data‐centric applications.^[^ ^21^, ^22^, ^23^ ^]^ When integrated with waveguides, GST‐based devices can break the diffraction limit for photonic memory^[^ ^24^ ^]^ and flexible displays^[^ ^25^ ^]^ with high bit density and non‐volatile features. Besides, electronic and photonic neuro‐inspired computing based on GST is now being actively developed.^[^ ^26^, ^27^, ^28^, ^29^, ^30^ ^]^ These advanced applications rely on a unique combination of material properties; the rapid and reversible phase transition between the crystalline and amorphous phase of GST, as well as the pronounced contrast in electrical and optical properties between the two phases.^[^ ^2^ ^]^ The strong property contrast has been explained by the change in bonding and conduction mechanisms upon phase transition.^[^ ^31^ ^]^

GeTe is a prototypical binary PCM system. It forms an ordered rhombohedral phase upon crystallization, which can be regarded as a distorted rocksalt structure with three short and three long Ge‐Te bonds. On average, the crystal possesses 2 *s* and 3 *p* valence electrons per atom, implying that the octet rule cannot be satisfied. Instead, crystalline GeTe is stabilized by a special bonding mechanism, the so‐called metavalent bonding (MVB), which involves mostly the *p* electrons and holds neighboring atoms together by one *p* electron (half an electron pair, i.e., a two‐center one‐electron bond).^[^ ^32^, ^33^, ^34^, ^35^, ^36^, ^37^ ^]^ This leads to more pronounced electron delocalization than in other semiconductors, such as crystalline Ge, which features a highly localized covalent bonding via *sp* ^3^ hybridization. When moving toward Sb_2_Te_3_ along the pseudo‐binary line, GST alloys also form a rocksalt‐like phase upon rapid crystallization, in which the excess *p* electrons brought by Sb are compensated by the high amounts of atomic vacancies in the Ge/Sb sublattice (e.g., 10% vacancies in Ge_2_Sb_2_Te_5_ and 16.67% in Sb_2_Te_3_),^[^ ^38^, ^39^, ^40^ ^]^ resulting in 3 *p* valence electrons per site on average. MVB is preserved in this rocksalt phase, giving rise to a high dielectric constant.^[^ ^32^ ^]^ The statistical distribution of these vacancies induces Anderson localization of electrons near the band edges.^[^ ^40^, ^41^, ^42^, ^43^ ^]^

Upon amorphization, bonding acquires covalent character due to the misalignment of *p* orbitals, inducing a drastic change in electrical and optical properties. This qualitative picture has recently been put on firm theoretical grounds by the analysis of bonding in terms of two fundamental quantum‐mechanical indicators, namely the electron transfer (ET) and the electrons sharing (ES) between pairs of neighboring atom.^[^ ^36^ ^]^ In addition to ET/ES values, a variety of property‐based fingerprints can be used to distinguish MVB from other bonding mechanisms, including the Born effective charge, effective coordination number, electrical conductivity, optical dielectric constant and Grüneisen parameters.^[^ ^31^ ^]^

Moreover, the delocalized bonding nature of MVB solids leads to an unconventional bond rupture phenomenon: upon laser‐assisted field evaporation in atom probe tomography (APT) experiments, atoms are dislodged from the surface of sample specimen mostly in a collective mode in crystalline PCMs, in stark contrast with amorphous PCMs, and other metallic, covalent or ionic solids, where atoms or ions are dislodged in single‐particle mode mostly.^[^ ^44^, ^45^, ^46^ ^]^ The difference in bonding character is further evidenced by an in‐depth ab initio analysis,^[^ ^47^ ^]^ and an unbiased classification algorithm,^[^ ^48^ ^]^ which classifies a number of about 330 solids into 4 different families, identified as metallic, ionic, covalent and metavalent solids. Recently, the projected phononic force‐constant tensors^[^ ^49^ ^]^ and atomic Hirshfeld surfaces^[^ ^50^ ^]^ are also suggested to be useful descriptors for screening of MVB solids.

In parallel, a hypervalent bonding mechanism is proposed instead to explain the novel phenomena in PCMs, which advocates an electron‐rich scheme (3 center – 4 electron).^[^ ^51^, ^52^ ^]^ Yet, a detailed quantum‐chemical bonding analysis shows that the bonding is electron‐deficient and not electron‐rich.^[^ ^53^ ^]^ A thorough discussion on why the electron‐deficient MVB scheme is essential to understand the unconventional bonding properties of crystalline PCMs can be found in a very recent review article.^[^ ^53^ ^]^

Although the (distorted) rocksalt phase is employed in PCM memories as one of the two logical states, it is in fact metastable, and further thermal annealing drives a vacancy ordering process,^[^ ^41^, ^54^ ^]^ triggering a gradual structural transition toward their ground state – an ordered trigonal (*t*‐) phase.^[^ ^55^, ^56^, ^57^ ^]^ The *t*‐phase consists of alternately stacked Te and Ge/Sb layers and is best visualized in the conventional hexagonal cell (in fact, it is often termed as hexagonal phase in the literature). For instance, Sb_2_Te_3_ consists of three Te‐Sb–Te‐Sb–Te quintuple‐layer (QL) blocks that are separated by three structural gaps. For GeSb_2_Te_4_ and Ge_2_Sb_2_Te_5_, their trigonal phase consists of septuple‐layer (SL) and nonuple‐layer (NL) blocks. These structural gaps are commonly regarded as van der Waals (vdW) gaps due to the long Te—Te interatomic distance over 3.7 Å across the gap, which breaks the network of closely connected *p* orbitals in the atomic blocks. Nevertheless, the trigonal phase shows several properties that have been linked to MVB, including a high dielectric constant and an unconventional collective‐bond‐rupture process in atom probe tomography experiments.^[^ ^46^ ^]^ The narrower gap size (as compared to 2D vdW materials) and the additional orbital overlap found in the gap region^[^ ^46^, ^58^ ^]^ suggest that the vdW gaps in these layered tellurides are nonpure.^[^ ^59^ ^]^

## 2 Results and Discussion

Here, we clarify how MVB develops across structural gaps in the trigonal phase of Sb_2_Te_3_ and GST alloys, and how MVB can be effectively tailored by stacking disorder and uniaxial strain for tunable dielectric and optical properties by carrying out in‐depth atomic imaging and optical experiments in combination with ab initio simulations. We focus on Sb_2_Te_3_ in the following as the structural gaps are most frequently encountered for a given film thickness in its trigonal phase. The data on trigonal GeSb_2_Te_4_ and Ge_2_Sb_2_Te_5_ are included in the Supporting Information. A Sb_2_Te_3_ thin film with a thickness of 300 nm was deposited on a silicon substrate via magnetron sputtering and was then annealed at 300 °C over 30 min to form the *t*‐Sb_2_Te_3_ phase. A capping layer was also deposited immediately afterward to prevent oxidation. As shown in Figure S1 (Supporting Information), the energy dispersive X‐ray (EDX) experiments showed the composition of the sputter thin films to be Sb_2_Te_3_. Clear structural features of trigonal Sb_2_Te_3_ were confirmed by the X‐ray diffraction (XRD) measurements. Subsequently, a specimen of the thin film was prepared with a thickness of about 80 nm for microstructure characterizations using the focused ion beam (FIB) system. The structural and chemical details of the *t*‐Sb_2_Te_3_ thin film were acquired by the high‐angle annular dark field (HAADF) imaging technique on a spherical aberration‐corrected scanning transmission microscope (STEM) equipped with an energy‐dispersive X‐ray (EDX) mapping analysis system. In contrast with the high‐quality thin films produced by epitaxial growth, structural defects are frequently created in *t*‐Sb_2_Te_3_ samples grown by sputtering.

**Figure 1:**
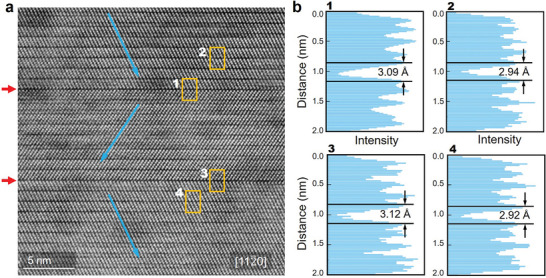
Atomic‐scale structural characterization. a) The HAADF image of trigonal Sb_2_Te_3_ thin film. Blue arrows denote different stacking sequences. Red arrows point at the boundaries (gaps) between the inverse stacking blocks. b) The image intensity profiles collected along the c‐axis for the boxed areas shown in (a), showing different widths of the gaps.

**Figure** **1**a shows a typical HAADF image of the sputtered *t*‐Sb_2_Te_3_ thin film. The bright dots in the HAADF image correspond to the positions of the atomic columns in the view direction [11$\bar{2}$0], and the intensity is approximately proportional to Z^2^, where Z represents the averaged atomic number of each column.^[^ ^60^ ^]^ As indicated by the blue arrows, the stacking sequence is reversed along the [0001] direction. Interestingly, at the boundaries between the inverse blocks (red arrows), the image contrast is slightly stronger (i.e., the gap area looks lightly darker) than at the gaps between the regular stacking blocks. A detailed analysis of the gap width in the areas marked by the yellow boxes, is shown in Figure 1b. We define the size of the gap (*d*_gap_) separating two QLs as the distance between the two relevant Te planes. The measurements of the image intensity profile collected along the vertical direction show that the width of the gap between the QLs with normal stacking is indeed smaller than that with inverse stacking (see also Figure S2, Supporting Information).

We made several HAADF scans in different areas of the sample and measured the size of the gaps, resulting in *d*_gap_ = 2.92±0.03 Å for the gap of the QLs with normal stacking and *d*_gap_ = 3.10±0.03 Å for the gap with inverse stacking. This increase in *d*_gap_ by ≈6.2% already indicates that the nature of the chemical interaction at the gaps is not “pure” vdW, for which the gap size should be insensitive to the stacking order. The latter behavior has indeed been observed in vdW materials containing ample stacking faults.^[^ ^61^ ^]^ We also measured the spacing between three normal QLs and QLs which include one inverse block, which gives a local *c* value of 30.39 ± 0.03 and 30.88 ± 0.03 Å, respectively. The averaged value over the whole thin film sample is *c* = 30.50 Å by XRD measurement (Figure S1, Supporting Information), which is slightly larger than the literature data, 30.46 Å, measured for single‐crystal bulk sample.^[^ ^62^ ^]^ This difference could be attributed to the presence of inverse blocks in the thin film sample. As directly measured on the HAADF image in the view direction [11$\bar{2}$0], the angle of Sb—Te—Te layers is 167±2^o^ at the interfaces between normal QLs, while it becomes 105±2^o^ at the inverse boundaries between the inverse blocks, indicating a clear misalignment of bond chains across the gap.

**Figure 2:**
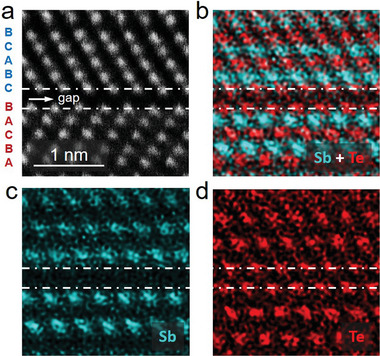
Element distribution analysis. a) A zoomed‐in HAADF image of an inverse stacking boundary (—A—B—C— vs —A—C—B—) and the corresponding b) overlaid and elemental EDX mappings, c) Sb – cyan and d) Te – pink.

**Figure** **2**a shows a zoom‐in HAADF image of the inverse blocks. The atomic layers take three special sites, which are arranged in —A—B—C— stacking along the vertical direction, but change to —A—C—B— stacking in the inverse block. Due to the very close atomic number of Sb (51) and Te (52), the intensity for the Sb and Te atomic columns looks almost the same in the HAADF image. EDX mapping provides direct chemical element distribution on the atomic scale as shown by the color maps in Figure 2. From the element maps, it is clear that inside each QL, three Te layers and two Sb layers are alternately stacked along the [0001] direction, and the compositional order is unchanged in the neighboring blocks having inverse stacking sequence. Similar stacking disorder was also observed in layer‐structured GeSb_2_Te_4_, Ge_2_Sb_2_Te_5_ and GeTe/Sb_2_Te_3_ superlattices.^[^ ^63^, ^64^, ^65^, ^66^, ^67^, ^68^, ^69^ ^]^ The compositional order between normal and inverse atomic blocks remains the same,^[^ ^66^ ^]^ and the enlarged interatomic spacing is consistently observed at the inverse stacking boundaries.^[^ ^67^ ^]^ This stacking fault is a consequence of vacancy‐ordering mediated structural transformation from the rocksalt phase,^[^ ^40^, ^70^ ^]^ which is absent in the high‐quality epitaxial grown thin film of *t*‐Sb_2_Te_3_ (Figure S3, Supporting Information).

**Figure 3:**
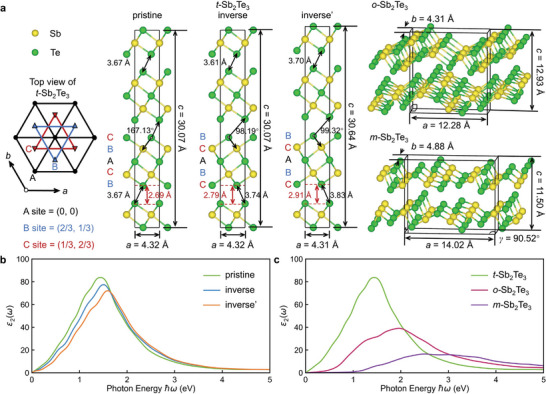
DFT calculations of Sb_2_Te_3_. a) From left to right: The top view of the three typical in‐plane atomic arrangements in trigonal Sb_2_Te_3_ (*t*‐Sb_2_Te_3_), the *t*‐Sb_2_Te_3_ with standard stacking sequence (pristine) and with an inverse block (inverse’ – fixed cell and inverse – relaxed cell) inside one unit cell, and the hypothetical models in orthorhombic (*o*‐Sb_2_Te_3_) and monoclinic (*m*‐Sb_2_Te_3_) structures. Yellow and green spheres correspond to Sb and Te atoms, respectively. b) The imaginary part (*ε*_2_) of the dielectric function of *t*‐Sb_2_Te_3_ with and without stacking disorder. c) The *ε*_2_ of *o*‐Sb_2_Te_3_ and *m*‐Sb_2_Te_3_ in comparison with pristine *t*‐Sb_2_Te_3_.

Next, we carried out density functional theory (DFT) calculations to gain a deeper understanding of the stacking disorder and how these defects can affect the bonding characters and physical properties of layered PCMs. The vdW interaction was treated with Grimme's D3 method;^[^ ^71^ ^]^ this and other technical details can be found in the Experimental Section. As shown in **Figure** **3**a, the in‐plane arrangement of the A, B and C atomic layers takes the three special positions of a hexagonal cell, namely, (0, 0), (2/3, 1/3) and (1/3, 2/3), respectively. Upon structural relaxation, the pristine *t*‐Sb_2_Te_3_ unit cell shows lattice parameters a = 4.32 Å and *c* = 30.07 Å, short and long Sb—Te bonds equal to 3.02 and 3.18 Å, a much longer interatomic distance of 3.67 Å for the Te—Te contacts across the structural gap and a gap size of 2.69 Å. The Sb—Te—Te motif formed by a Sb—Te bond and a neighboring Te—Te contact has a bond angle of 167.13°, which is comparable to the bond angle between quasi‐aligned atomic pairs inside the QL.

If we keep the lattice parameters fixed but include one inverse block and relax the atomic coordinates, we notice a drastic change in Sb—Te—Te bond angle to 98.19° and a small increase in the Te—Te contact *d*_TT_ to 3.74 Å and in *d*_gap_ to 2.79 Å at the boundary of the inverse block. If we allow the simulation cell to relax, the *c* edge increases by 0.57 Å, due mainly to the significantly longer Te—Te contact *d*_TT_ = 3.83 Å (*d*_gap_ = 2.91 Å) at the inverse boundary. The models with unrelaxed and relaxed simulation cell are denoted with inverse and inverse’ in Figure 3a. Such increased width of the inverse boundary gap agrees well with the HAADF images shown in Figure 1. The total energy of the inverse and inverse’ models are about 4.0 and 2.9 meV per atom higher than the pristine model. This reduction in total energy obtained by increasing the interatomic spacing explains why wider structural gaps are observed at the inverse stacking boundaries in the HAADF images. Projecting the Sb—Te—Te bond angle according to the [11$\bar{2}$0] view direction, the angle between the Sb—Te—Te layers is 168.63^o^ between normal QLs in the pristine model, but 103.38^o^ at the inverse stacking boundary in the inverse’ model, consistent with the STEM values. All structural details of the trigonal models are summarized together with experimental data in **Table** **1**. Additional calculations using other functional^[^ ^72^ ^]^ and vdW corrections^[^ ^73^, ^74^ ^]^ were also considered. Overall, PBE+D3 calculations yield a better comparison with STEM experiments, and we stick to this combination for the following discussions.

**Table 1:** Structural details of pristine and defective *t*‐Sb_2_Te_3_ models. The lattice parameter *c*, Te−Te interatomic distance *d*_TT_, gap size *d*_gap_ and Sb−Te−Te bond angle across the gap are calculated with different combination of functional and vdW corrections

*t*‐Sb_2_Te_3_	parameters	STEM exp.	PBE+D3	PBE+TS	PBE+D2	PBE	PBESol	PBESol+D3
pristine inverse'	*c* (Å)	30.39±0.03 30.88±0.03	30.07 30.64	30.01 30.83	30.93 31.16	31.36 31.91	29.84 30.34	29.23 29.79
pristine inverse’	*d*_TT_ (Å)	/	3.67 3.83	3.68 3.93	3.81 3.87	3.96 4.13	3.6 3.74	3.47 3.62
pristine inverse’	∠Sb−Te−Te (°)	/	167.1 99.3	167.0 99.98	165.1 100.9	163.5 101.5	167.95 99.27	169.8 98.3
pristine inverse’	*d*_gap_ (Å)	2.92±0.03 3.10±0.03	2.69 2.91	2.7 3.04	2.92 3.0	3.06 3.29	2.62 2.82	2.45 2.67
/	Δ*d*_gap_	≈6.2%	8.18%	12.59%	2.74%	7.52%	7.63%	8.98%

In order to draw a comparison with standard vdW materials, we perform similar calculations for crystalline SnSe_2_ and SiTe_2_ in the 1T phase, where each atomic block is made of three atomic layers in an octahedral arrangement. In stark contrast with *t*‐Sb_2_Te_3_, the changes in lattice parameter and total energy upon inclusion of inverse blocks (Figure S4, Supporting Information) are exceedingly small in 1T‐SnSe_2_ and 1T‐SiTe_2_ (below 0.02 Å per unit block and 0.8 meV per atom). This difference further illustrates that Sb_2_Te_3_ cannot be regarded as a vdW material. Trigonal GST alloys exhibit similar behavior as Sb_2_Te_3_ with a clear increase in gap size at the inverse stacking boundaries (Figure S5, Supporting Information). Hence, the structural gaps in these layered phase‐change tellurides should be referred as vdW‐like gaps instead vdW gaps.

We also evaluate the impact of the inverse block on the dielectric properties of trigonal Sb_2_Te_3_ and GST. Figure 3b shows the imaginary part (*ε*_2_) of the dielectric function for pristine, inverse and inverse’ *t*‐Sb_2_Te_3_ (the real part *ε*_1_ is shown in Figure S6, Supporting Information). A reduction in the *ε*_2_ values is clearly observed below 1.6 eV if the bond alignment across the vdW‐like gaps is broken (inverse structure). *ε*_2_ further decreases when the Te—Te interatomic distance is increased (inverse’ structure). The decrease is accompanied by a gradual blue‐shift of the peak value. The difference in *ε*_2_ between pristine and inverse’ *t*‐Sb_2_Te_3_ amounts to a ≈14% reduction in the peak value: this is significant but is much smaller than the peak‐value difference between trigonal and amorphous Sb_2_Te_3_, ≈63% (see Ref. [75] and the following discussion), because in the inverse’ models the Sb—Te bonds are still highly aligned and closely coupled inside each atomic block, including the inverse one.

In the amorphous model, high angular disorder is present, breaking the alignment of *p* orbitals at short interatomic distance. Here, we considered two additional hypothetical structures to break the bond alignment inside the atomic blocks. Specifically, we computed the structural and optical properties of Sb_2_Te_3_ taking the other two frequently observed phases of pnictogen sesqui‐chalcogenides, namely, the orthorhombic Sb_2_Se_3_ structure and the monoclinic As_2_Se_3_ structure. The relative atomic coordinates were kept unchanged, while the cell volume was relaxed to reduce the internal stresses. The hypothetical structures obtained are shown in Figure 3a (right panel) and are denoted as *o*‐ and *m*‐Sb_2_Te_3_. If we set a large cutoff of 3.4 Å for Sb—Te bonds, we observe aligned Te‐Sb–Te‐Sb–Te chains in *o*‐Sb_2_Te_3_, however, there is no alignment through the weak atomic contacts across the zigzag gaps. As shown in Figure 3c, its dielectric function is strongly weakened with a large reduction in the peak value of ≈53% with respect to *t*‐Sb_2_Te_3_. In *m*‐Sb_2_Te_3_, the bond alignment for Sb—Te bonds is completely absent, resulting in very low values of *ε*_2_, which is even weaker than the one of the amorphous phase.^[^ ^75^ ^]^

**Figure 4:**
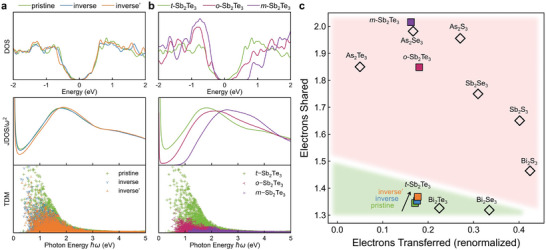
Electronic structure and bonding calculations. The density of states (DOS), joint density of states (JDOS) and transition dipole moments (TDM) for a) pristine, inverse’ and inverse *t*‐Sb_2_Te_3_ and b) *o*‐Sb_2_Te_3_ and *m*‐Sb_2_Te_3_ in comparison with pristine *t*‐Sb_2_Te_3_. c) The bonding map for Sb_2_Te_3_ in the five atomic configurations listed in Figure 3a and for other pnictogen sesqui‐chalcogenides (i.e., V_2_VI_3_, V = As, Sb, Bi, VI = S, Se, Te) in their ground state. The two coordinates of the map are the Electron Transferred (ET) and the Electrons Shared (ES) values between adjacent atoms. The green and red shaded regions indicate the domain of metavalent bonding and of covalent bonding, respectively.

To gain a better understanding of the difference in dielectric functions of these models, we performed electronic structure and chemical bonding analyses. As shown in **Figure** **4**a, the overall DOS shape of the pristine and defective *t*‐Sb_2_Te_3_ models is similar, except for some small numerical differences around the energy gap. The profile of *ε*_2_ is composed of the joint density of states (JDOS) and the transition dipole moments (TDMs), accounting for the density of possible inter‐band excitations and the transition probability for each excitation, respectively.^[^ ^76^ ^]^ The JDOS profiles almost overlap for the pristine and inverse model, yet the inverse’ model shows lower JDOS values below 1.6 eV. Regarding the TDMs, the pristine model shows consistently higher values than the inverse and inverse’ models between 0.5 and 1.6 eV. Therefore, the transition probabilities for the relevant excitations are reduced if the weak‐bond alignment across the vdW‐like gaps is broken (inverse *t*‐Sb_2_Te_3_), thus decreasing *ε*_2_. This is in line with the fact that, generally, a reduction in the overlap of the wave functions involved in the optical transitions leads to a decrease of the corresponding transition probabilities.^[^ ^76^ ^]^ Much larger differences in DOS, JDOS and TDM profiles are observed between pristine *t*‐Sb_2_Te_3_ and the two hypothetical structures (Figure 3b), leading to a wider contrast window in *ε*_2_.

We also calculated the ET and ES values (see Experimental Section) to characterize the bonding differences between the Sb_2_Te_3_ models discussed above (see Figure 4c). We also considered other sesqui‐chalcogenides (i.e., V_2_VI_3_, V = As, Sb or Bi; VI = S, Se or Te) in their ground state for comparison.^[^ ^46^ ^]^ The detailed atomic structures and lattice parameters of the latter compounds are included in Figure S7 (Supporting Information). We used the normalized ET value, i.e., the electron transfer divided by the formal oxidation state of the respective atom, and the ES value of the shortest bond in each model. Consistent with literature, three alloys, namely, Sb_2_Te_3_, Bi_2_Te_3_ and Bi_2_Se_3_ in the trigonal structure, are located in the MVB region (marked in green), while other sesqui‐chalcogenides appear in the covalent bonding (CVB) region (marked in pink). The alloys located in the CVB region show distinct properties with respect to the MVB alloys, and the bond rupture of the former proceeds mostly via single‐ion evaporation instead of multiple‐ions in APT experiments.^[^ ^44^, ^46^ ^]^ If a hypothetical trigonal phase is considered for Sb_2_Se_3_, it may also display MVB features with a stronger dielectric function than the orthorhombic phase (see Figure S8, Supporting Information). Nevertheless, it is more difficult for lighter pnictogen sesqui‐chalcogenides to form the trigonal phase, because these alloys show a stronger *sp* ^3^ mixing that competes with the *p* orbital dominant MVB mechanism. This change in bonding tendency stems from the smaller energy separation between the valence shell *s* and *p* orbitals in lighter elements.

From pristine to inverse and to inverse’ *t*‐Sb_2_Te_3_, a gradual change in ET and ES values toward the boundary between MVB and CVB is observed, indicating gradually weakened MVB. Regarding *o*‐ and *m*‐Sb_2_Te_3_, their ET and ES values are clearly located in the CVB region. We conclude that the breaking of the alignment of both the weakly coupled Te—Te contacts and the strongly coupled Sb—Te bonds undermines MVB, leading to smaller TDMs and a reduction in the dielectric function peak. Our dielectric function and bonding calculations also confirm the presence of non‐negligible orbital overlap in addition to the vdW forces across the gap regions in pristine *t*‐Sb_2_Te_3_, supporting previous experimental observations.^[^ ^46^, ^59^ ^]^ More specifically, in this region, there is no electron transfer between the weakly coupled Te atoms, but there exists a non‐negligible amount of shared electrons, denoted as ES_gap_. Indeed, in pristine *t*‐Sb_2_Te_3_, ES_gap_ equals 0.37 e, which is larger than previous calculations^[^ ^46^ ^]^ because of the shorter Te—Te interatomic distance, 3.67 Å, obtained by our DFT calculations upon inclusion of vdW corrections. This finite charge mediates the development of MVB across the structural gaps, and its value varies with the Te—Te interatomic distance, largely influencing the dielectric properties. Indeed, in the inverse and inverse’ models, ES_gap_ is reduced to 0.33 and 0.29 e, and the Te—Te contact length is increased to 3.74 and 3.83 Å, respectively.

**Figure 5:**
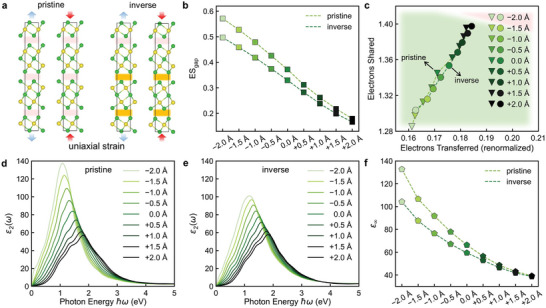
Uniaxial strain calculations. a) The sketch of uniaxial strain applied in pristine and inverse *t*‐Sb_2_Te_3_. Both tensile (blue arrows) and compressive (red arrows) strain along the vertical direction are considered. The gap regions separating blocks without and with stacking inversion are marked by pink and orange, respectively. The changes in b) the shared electrons in the gap region – ES_gap_ and c) the ET and ES values of the short Sb—Te bonds, the *ε*_2_ profiles for d) pristine and e) inverse *t*‐Sb_2_Te_3_ and f) the dielectric constant *ε*_∞_ under uniaxial strain.

Finally, uniaxial strain was applied to the *c* axis to tailor ES_gap_ further, as sketched in **Figure** **5**a. For the sake of convenience, we only considered the pristine and inverse models, which have the same set of lattice parameters, for the strain calculations. We kept the *a*‐ and *b*‐edge fixed, while increasing and decreasing the *c*‐edge from −2.0 to +2.0 Å, corresponding to a change of less than ±7% lattice strain. As shown in Figure 5b, the ES_gap_ value increases upon compressive strain and decreases upon tensile strain for both the pristine and inverse *t*‐Sb_2_Te_3_ models. The difference in ES_gap_ between the pristine and inverse models gets larger at −2.0 Å because of the stronger overlap (the Te—Te interatomic distance reaches 3.37 Å) and better alignment of *p* orbitals (the Sb—Te—Te bond angle reaches 171°) in the pristine model (see more structural details in **Table** **2**). The ES_gap_ value is largely reduced to around 0.17 e for both models under tensile strain at +2.0 Å, and the Te—Te interatomic distance is increased to above 4.1 Å. The strain effects also induce changes in the ET and ES values for the atoms inside the atomic blocks. As shown in Figure 5c, compressive strain makes the bonding more MVB‐like, while tensile strain drives the system toward the CVB region.

**Table 2:** Strain calculations. The structural details of the pristine *t*‐Sb_2_Te_3_ model under uniaxial compressive and tensile strain

pristine *t*‐Sb_2_Te_3_	−2.0 Å	−1.0 Å	0.0 Å	+1.0 Å	+2.0 Å
*c* (Å)	28.07	29.07	30.07	31.07	32.07
*d*_TT_ (Å)	3.37	3.5	3.67	3.89	4.14
*d*_gap_ (Å)	2.27	2.45	2.69	2.99	3.3
∠Sb−Te−Te (°)	171.4	169.6	167.1	164.2	161.4

The change in bonding character and in overlap between the *p* orbitals has direct impact on the dielectric properties, since it strongly affects the optical transitions. As shown in Figure 5d,e, a systematic reduction in *ε*_2_ from −2.0 to +2.0 Å is observed in both pristine and inverse *t*‐Sb_2_Te_3_ below 1.9 eV, accompanied by a blue‐shift of the peak value. The tailoring of *ε*_2_ upon straining is clearly more effective in the pristine model due to the bond alignment across the vdW‐like gaps. The same trend holds for *ε*_1_ (Figure S6, Supporting Information) and *ε*_∞_ (Figure 5f). The compressive strain results in a large increase in *ε*_∞_ from 66 at equilibrium to 132 when the *c*‐edge is reduced by 2.0 Å in pristine *t*‐Sb_2_Te_3_, due to the enhanced MVB and increased ES_gap_. Note that the total energy also rises, because the edge Te atoms are all negatively charged, inducing stronger electrostatic repulsion when getting closer. Nevertheless, the increase in total energy in the pristine model, 24 meV per atom (−2.0 Å), is lower than that in the inverse model, 40 meV per atom (−2.0 Å), indicating the former bonding scenario to be more comfortable. When the *c*‐edge is stretched at +2.0 Å, there is hardly any difference in *ε*_∞_, *ε*_1_ and *ε*_2_ between the pristine and inverse model, because MVB is broken across the gaps due to the large interatomic distance over 4.1 Å. In this limit, only the aligned Sb—Te bonds inside each QL contribute to the dielectric function.

**Figure 6:**
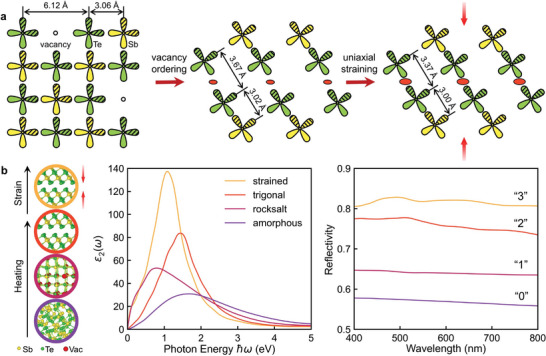
Bonding mechanisms and optical properties. a) The schematic overview of bonding mechanisms in crystalline Sb_2_Te_3_ upon heating and straining. b) Atomic structures of amorphous, rocksalt, trigonal Sb_2_Te_3_ and *t*‐Sb_2_Te_3_ under compressive strain (−2.0 Å) and the corresponding *ε*_2_ and optical reflectivity *R* by DFT calculations. Four distinct levels in reflectivity are found.

Before closing, we provide an overview about the change in bonding character and optical response upon phase transition in Sb_2_Te_3_. Upon heating, the amorphous phase first crystallizes into the rocksalt‐like phase with a change in bonding mechanism from CVB to MVB. As shown in **Figure** **6**a, all the Sb—Te octahedral bonds are strongly coupled, but the alignment of *p* orbitals could be broken when encountering atomic vacancies, which occupy 1/3 of the Sb sublattice. The interatomic distance between two Te atoms separated by a vacancy is over 6.12 Å, and no shared electron exists over such long distance. Further thermal annealing drives a vacancy ordering process, inducing a structural transformation into the trigonal phase. The high amount of atomic vacancies segregates into 2D vacant layers, resulting in shorter Te—Te interatomic distances across the gap as compared to the previous case. The shared electrons between the weakly coupled Te atoms enable MVB between QLs, which can be further strengthened by adding uniaxial compressive strain. The four configurations, namely, the amorphous, rocksalt, trigonal and strained trigonal (−2.0 Å) Sb_2_Te_3_, indeed display well‐separated *ε*_2_ and optical reflectivity *R* (Figure 6b). Our optical measurements of Sb_2_Te_3_ thin films annealed at different temperatures confirmed that trigonal phase indeed has a higher reflectivity than the rocksalt phase (Figure S9, Supporting Information). Inverted stacking sequence and uniaxial strain have similar effects on other layered PCMs, including GeSb_2_Te_4_ and Ge_2_Sb_2_Te_5_ (Figure S10, Supporting Information). Note that the calculated optical contrast between amorphous and rocksalt Sb_2_Te_3_ is smaller than that of GeSb_2_Te_4_,^[^ ^70^ ^]^ which can be attributed to the larger number of “broken bonds” in rocksalt Sb_2_Te_3_ due to the larger concentration of vacancies in the cation‐like sublattice, namely 1/3 versus 1/4 vacant sites in rocksalt Sb_2_Te_3_ and GeSb_2_Te_4_, respectively. This observation is consistent with previous optical measurements, showing a reduction in Δ*R* upon crystallization for increasing concentration of vacancies in the crystalline state.^[^ ^77^ ^]^

## 3 Conclusion

In summary, we have carried out atomic imaging experiments and ab initio calculations to gain an in‐depth understanding on the structural and bonding nature of the inverted stacking disorder in trigonal Sb_2_Te_3_. We have elucidated the importance of electron sharing between the edge Te atoms across the gaps for the existence of MVB along the vertical direction, and we have clarified how MVB is affected by the breaking of *p* orbital alignment across the gaps. The degree of MVB was further tailored by adding uniaxial strain perpendicular to the vdW‐like gaps, thus enabling a systematic tuning of the optical properties in the trigonal phase and potentially widening the programming window between the amorphous and rocksalt phase for optical and photonic PCM applications that require multilevel programming capacities. Nevertheless, the tradeoff on programming speed, power consumption and manufacturing complexity (for the implementation of mechanical forces) need to be carefully assessed when utilizing the trigonal phase. It may also be feasible to exploit the large variation in dielectric function in MVB alloys induced by strain for optical applications, i.e., the elastic strain engineering approach coupled with microelectromechanical systems,^[^ ^78^, ^79^, ^80^ ^]^ or the thin film bending approach using flexible substrates.^[^ ^81^ ^]^ Our suggestion for such use is to grow high‐quality thin films to minimize the presence of inverse blocks, allowing tuning of optical properties more effectively. The bonding mechanism and optical characters in layered chalcogenides could be further explored by evaluating the thickness‐dependent properties in ultrathin films^[^ ^82^, ^83^, ^84^, ^85^, ^86^ ^]^ or in the alternately grown heterostructure thin films, such as the TiTe_2_/Sb_2_Te_3_ heterostructure.^[^ ^87^, ^88^, ^89^, ^90^ ^]^ At last, we note that presence of the inverse block defects should also affect the electronic and thermal conduction like other extended defects, such as swapped bilayers^[^ ^91^ ^]^ and stacking faults with non‐QL blocks,^[^ ^92^ ^]^ which could be tailored for thermoelectric^[^ ^93^, ^94^ ^]^ and topological^[^ ^92^, ^95^ ^]^ applications of trigonal Sb_2_Te_3_ and related chalcogenides.

## 4 Experimental Section

### Magnetron Sputtering

The Sb_2_Te_3_ thin films were deposited using a stoichiometric Sb_2_Te_3_ alloy target by radio frequency voltage source in the AJA Orion‐8 sputtering system. The power is ≈50 W, and the deposition rate is ≈6.7 nm min^−1^. The films with the thickness of ≈300 and ≈450 nm were prepared on a pure Si substrate and a Si substrate covered with Pt layer of ≈120 nm, respectively. The thin films were then annealed at 80 °C and 300 °C over 30 min under high vacuum of ≈10^−8^ torr to form the rocksalt and trigonal phase, respectively.

### Molecular Beam Epitaxy

The Sb_2_Te_3_ thin film of ≈150 nm was deposited in the MBE Chamber at a base pressure of about 10^−11^ mbar (10^−10^ mbar during growth). The Si (111) substrate was passivated by Sb to obtain high‐quality thin film, and the deposition rate was 0.2 nm min^−1^. The sources were elemental Sb and Te effusion cells.

### Structural Characterizations

The thickness and composition of the films were measured by a Hitachi SU8230 SEM equipped with a Bruker QUANTAX ESPRIT 2 XFlash7 Energy Dispersive X‐ray spectrometer (EDX). The structure of the annealed thin films was determined by X‐ray diffraction (XRD) measurements using a setup of Bruker D8 ADVANCE, the range of 2*θ* was set between 10 to 60°. The cross‐section TEM specimen was prepared by a dual beam focused ion beam (FIB) system (Helios NanoLab 600i, FEI) with a Ga ion beam at 30 kV and polished at 5 kV to remove potential damage on the surface of the lamellar. The atomic resolution STEM–HAADF imaging experiments and EDX mapping analyses were performed on a Hitachi HF5000 environmental aberration‐corrected electron microscope equipped with a probe aberration corrector and an Oxford Instruments X‐MaxN 100 TLE spectrometer, operated at 200 keV.

### Optical Measurements

The optical measurements were performed with the thin film deposited on Pt substrate to prevent potential absorption from Si substrate. The reflection of the films was measured by the Ocean Optics‐HDX01528 spectrograph equipped with the HL‐2000‐FHSA Tungsten halogen light source. The wavelength range was set from 390 to 760 nm.

### Ab Initio Calculations

DFT calculations were carried out using the VASP code^[^ ^96^ ^]^ with the projector augmented‐wave (PAW) pseudopotentials,^[^ ^97^ ^]^ the Perdew–Burke–Ernzerhof (PBE) functional^[^ ^98^ ^]^ and Grimme's D3 dispersion correction.^[^ ^71^ ^]^ Other functional^[^ ^72^ ^]^ and vdW corrections^[^ ^73^, ^74^ ^]^ resulted in a similar trend in lattice expansion and reduction in dielectric function with the inclusion of inverse block defects, as presented in Table 1 and Figure S11 (Supporting Information). Trigonal Sb_2_Te_3_, GeSb_2_Te_4_ and Ge_2_Sb_2_Te_5_ were all modeled in their hexagonal unit cell or supercell. The amorphous Sb_2_Te_3_ was generated using VASP following a standard melt‐quench protocol,^[^ ^99^ ^]^ and the rocksalt Sb_2_Te_3_ was built in a 3×3×3 supercell, where the distribution of vacancies was generated using a random number generator. Both sets of models contained 180 atoms. The frequency‐dependent dielectric matrix was calculated within the independent‐particle approximation using VASP, which was shown to be adequate to account for the optical contrast between crystalline and amorphous PCMs.^[^ ^100^, ^101^, ^102^, ^103^ ^]^ The relaxed structures were calculated with the Quantum ESPRESSO code,^[^ ^104^ ^]^ which provided ground‐state wave‐functions for the bonding analysis using the Critic2 code.^[^ ^105^ ^]^ The Critic2 code calculates the domain overlap matrices (DOM) over Bader's basins, and the delocalization or localization indices (DIs/LIs) among or within such basins measure the quantity of electrons being localized in the atomic basin or shared between the two atoms.

### Statistical Analysis

The statistical analysis for STEM measurements is provided in the main text and the Supporting Information, wherever applicable.

## Conflict of Interest

The authors declare no conflict of interest.

## Supporting information

Supporting Information

## Data Availability

The data that support the findings of this study are available from the corresponding author upon reasonable request.
