# Supplementary material for: Metavalent Bonding in Layered Phase‐Change Memory Materials
Source: Adv Sci (Weinh). 2023 Mar 30;10(15):2300901. doi: 10.1002/advs.202300901 (PMC10214272; doi:10.1002/advs.202300901)
Supplement: Supplementary file 1 — Supporting Information [file ADVS-10-2300901-s001.pdf]

ADVANCED SCIENCE
Open Access

Supporting Information

for *Adv. Sci.*, DOI 10.1002/advs.202300901

Metavalent Bonding in Layered Phase-Change Memory Materials

Wei Zhang, Hangming Zhang, Suyang Sun, Xiaozhe Wang, Zhewen Lu, Xudong Wang, Jiang-Jing Wang*, Chunlin Jia, Carl-Friedrich Schön, Riccardo Mazzarello, En Ma and Matthias Wuttig**

Supporting Information

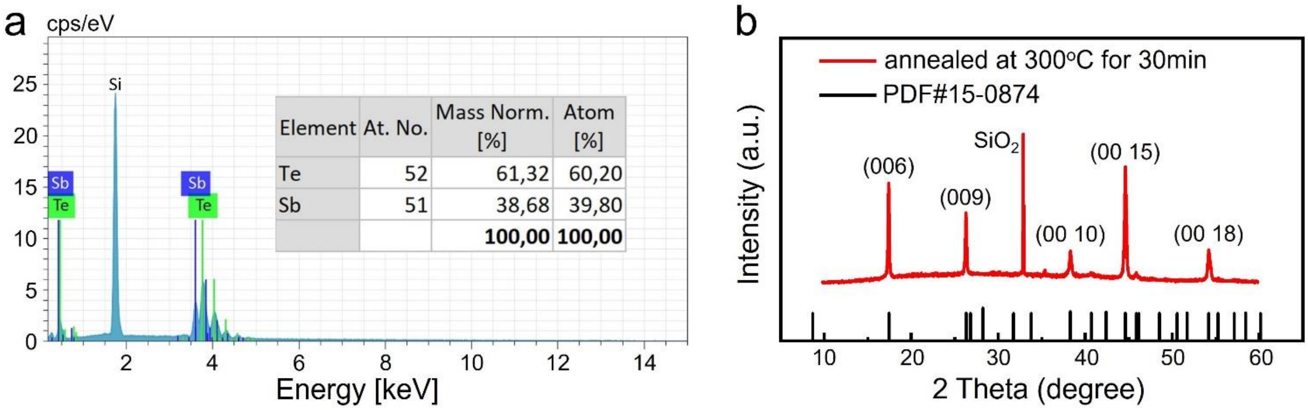

Figure S1. The energy dispersive X-ray measurement (EDX) and the X-ray diffraction (XRD) measurements of sputtered Sb_2Te_3 films.

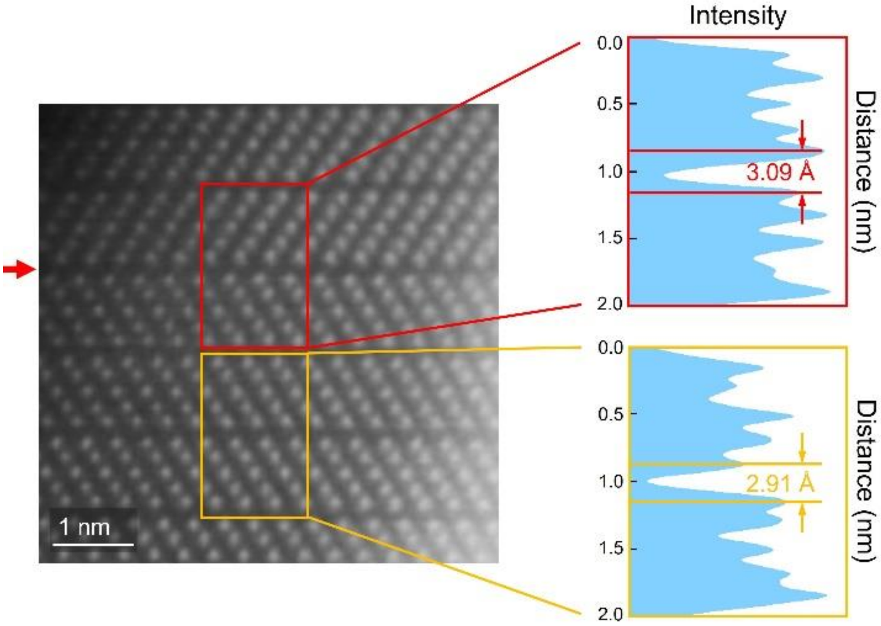

Figure S2. A HAADF image of the annealed *t*-Sb_2Te_3 thin film sample with image intensity analysis of the structural gaps.

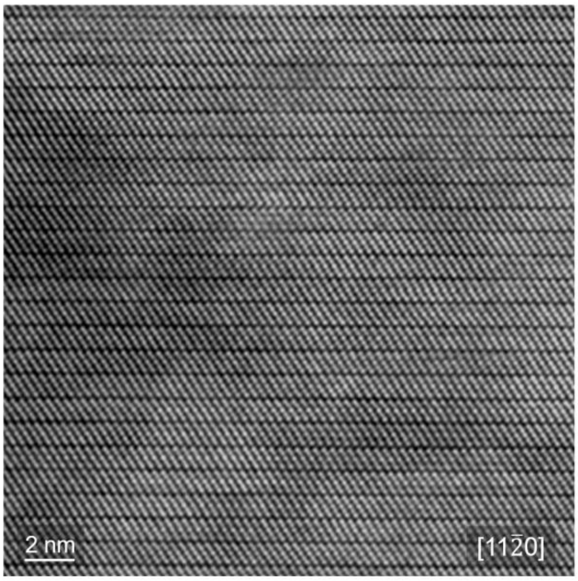

Figure S3. A HAADF image of *t*-Sb_2Te_3 thin film sample prepared by molecular beam epitaxy (MBE). No inverse blocks can be observed.

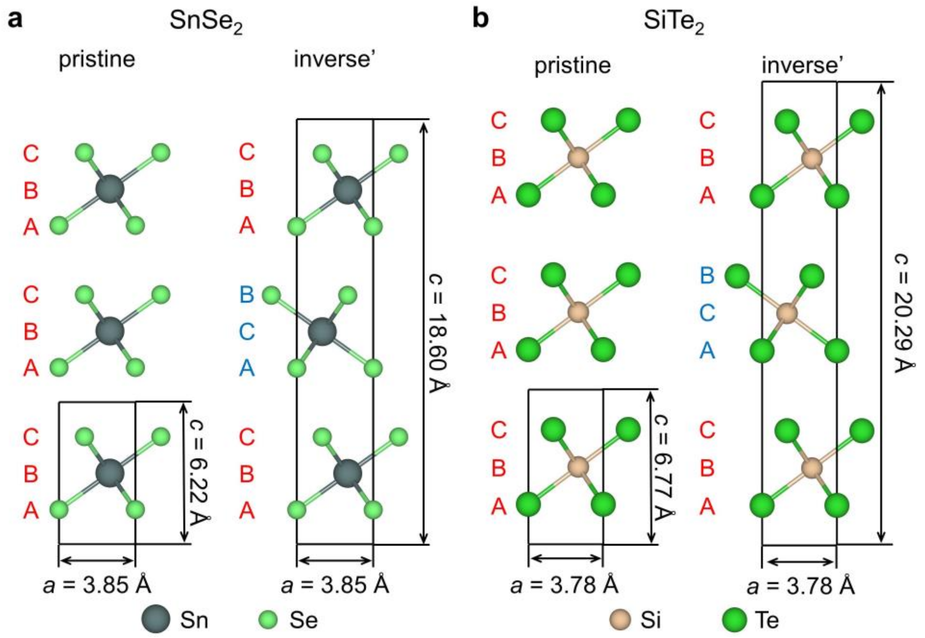

Figure S4. DFT-optimized structures of (a) 1T-$SnSe_2$ and (b) 1T-$SiTe_2$ with and without inverse block. The 1T-$SnSe_2$ / 1T-$SiTe_2$ unit cell was repeated three times along the vertical direction, and the stacking sequence of the middle block was reversed. Upon cell relaxation (with the inclusion of vdW forces by Grimme's D3 method), their *c* edge decreases very slightly. Per unit block, the change in lattice is below 0.02 Å. The change in total energy is also very small, less than 0.8 meV per atom.

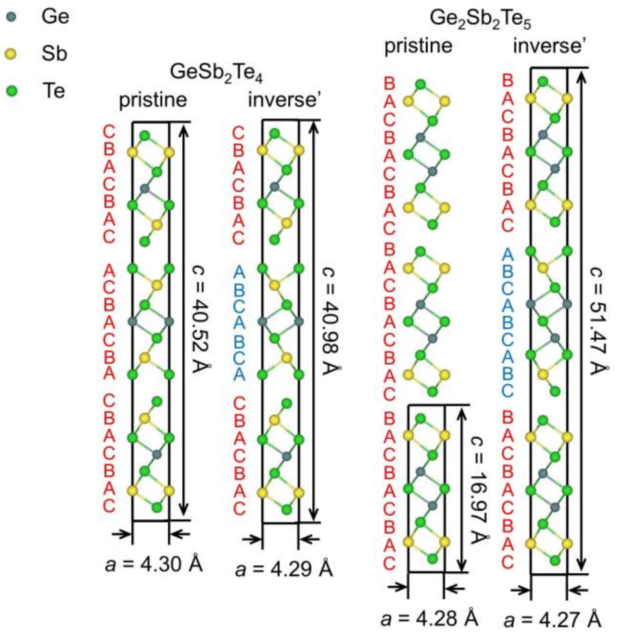

Figure S5. DFT-optimized structures of $GeSb_2Te_4$ and $Ge_2Sb_2Te_5$ with and without inverse block. Upon cell relaxation, the *c* edge increases from 40.52 Å (pristine structure) to 40.98 Å (inverse' structure) in *t*-$GeSb_2Te_4$. To model defective *t*-$Ge_2Sb_2Te_5$, the unit cell was repeated along the vertical direction by three times (*c* = 50.91 Å) and the stacking sequence of the middle block was reversed. Upon cell relaxation, the c edge increases to 51.47 Å.

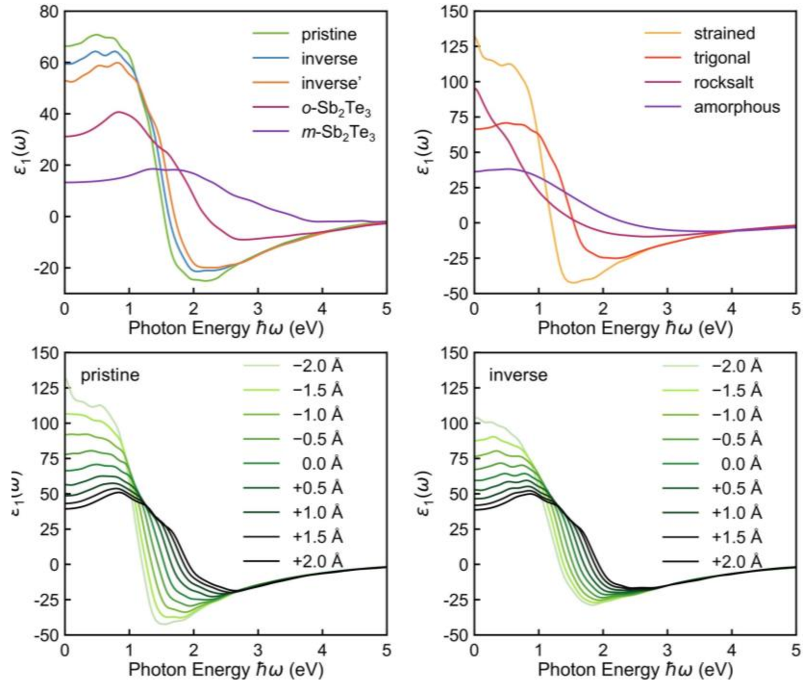

Figure S6. The real part (ε_1) of the dielectric function of Sb_2Te_3 for the structural phases considered in the main text. We note that the size of the band gap is underestimated using DFT-PBE, especially for the highly disordered systems. Hence, the values of ε_1 for the rocksalt and amorphous models at small phonon energies, corresponding to the spectral region near the energy gap, are not accurate.

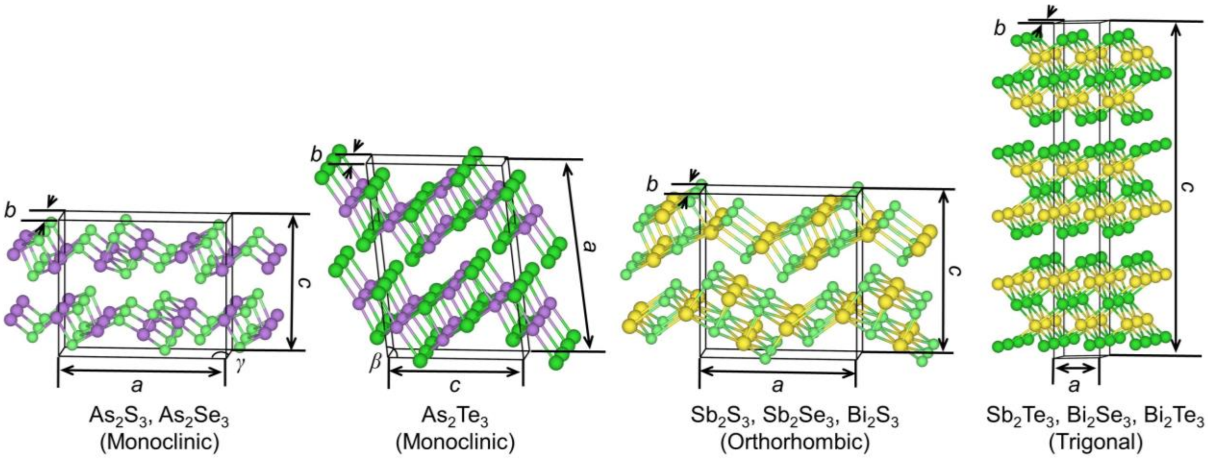

Ground-state structure	As	Sb	Bi
**S**	Monoclinic<br>*a* = 11.53 Å, *b* = 4.29 Å, *c* = 9.72 Å<br>α = 90°, β = 90°, γ = 90.50°	Orthorhombic<br>*a* = 11.46 Å, *b* = 3.86 Å, *c* = 11.09 Å<br>α = 90°, β = 90°, γ = 90°	Orthorhombic<br>*a* = 11.33 Å, *b* = 4.00 Å, *c* = 11.09 Å<br>α = 90°, β = 90°, γ = 90°
**Se**	Monoclinic<br>*a* = 12.23 Å, *b* = 4.26 Å, *c* = 10.03 Å<br>α = 90°, β = 90°, γ = 90.52°	Orthorhombic<br>*a* = 12.04 Å, *b* = 4.02 Å, *c* = 11.46 Å<br>α = 90°, β = 90°, γ = 90°	Trigonal<br>*a* = 4.17 Å, *b* = 4.17 Å, *c* = 28.94 Å<br>α = 90°, β = 90°, γ = 120°
**Te**	Monoclinic<br>*a* = 14.35 Å, *b* = 4.07 Å, *c* = 9.93 Å<br>α = 90°, β = 96.34°, γ = 90°	Trigonal<br>*a* = 4.32 Å, *b* = 4.32 Å, *c* = 30.07 Å<br>α = 90°, β = 90°, γ = 120°	Trigonal<br>*a* = 4.43 Å, *b* = 4.43 Å, *c* = 30.46 Å<br>α = 90°, β = 90°, γ = 120°

Figure S7. The atomic structures of V_2VI_3 alloys in their ground state optimized with DFT calculations. The Grimme's D3 vdW correction was included for all these calculations. The MVB alloys are marked in green, while the CVB alloys are marked in pink.

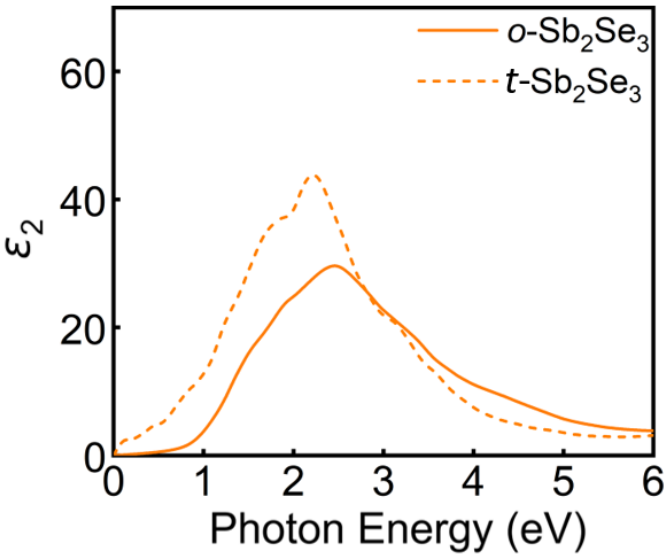

Figure S8. The DFT-calculated $\varepsilon_2(\omega)$ for *o*-Sb_2Se_3 and hypothetical *t*-Sb_2Se_3. The latter shows a stronger dielectric function than the former.

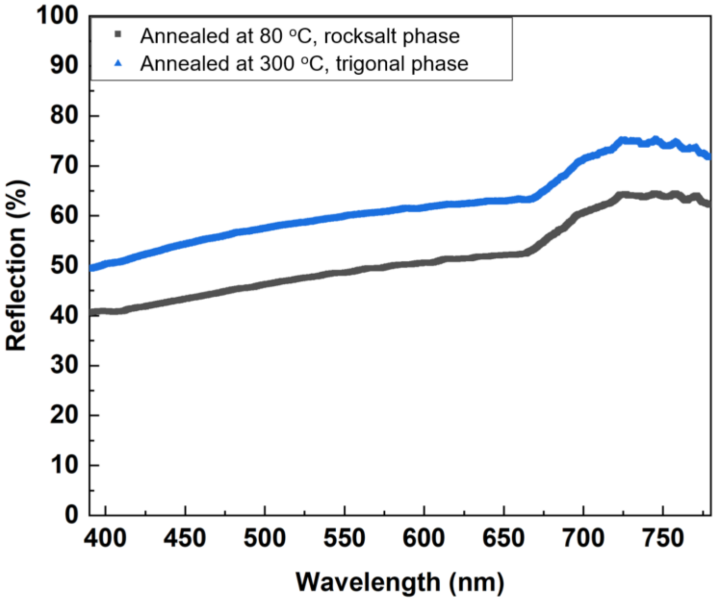

Figure S9. Optical reflectivity measured for rocksalt and trigonal Sb_2Te_3. The thin films were deposited on Pt substrate, and were annealed at 80 ^{o}C and 300 ^{o}C for half an hour. Their XRD patterns confirmed the formation of rocksalt and trigonal phase.

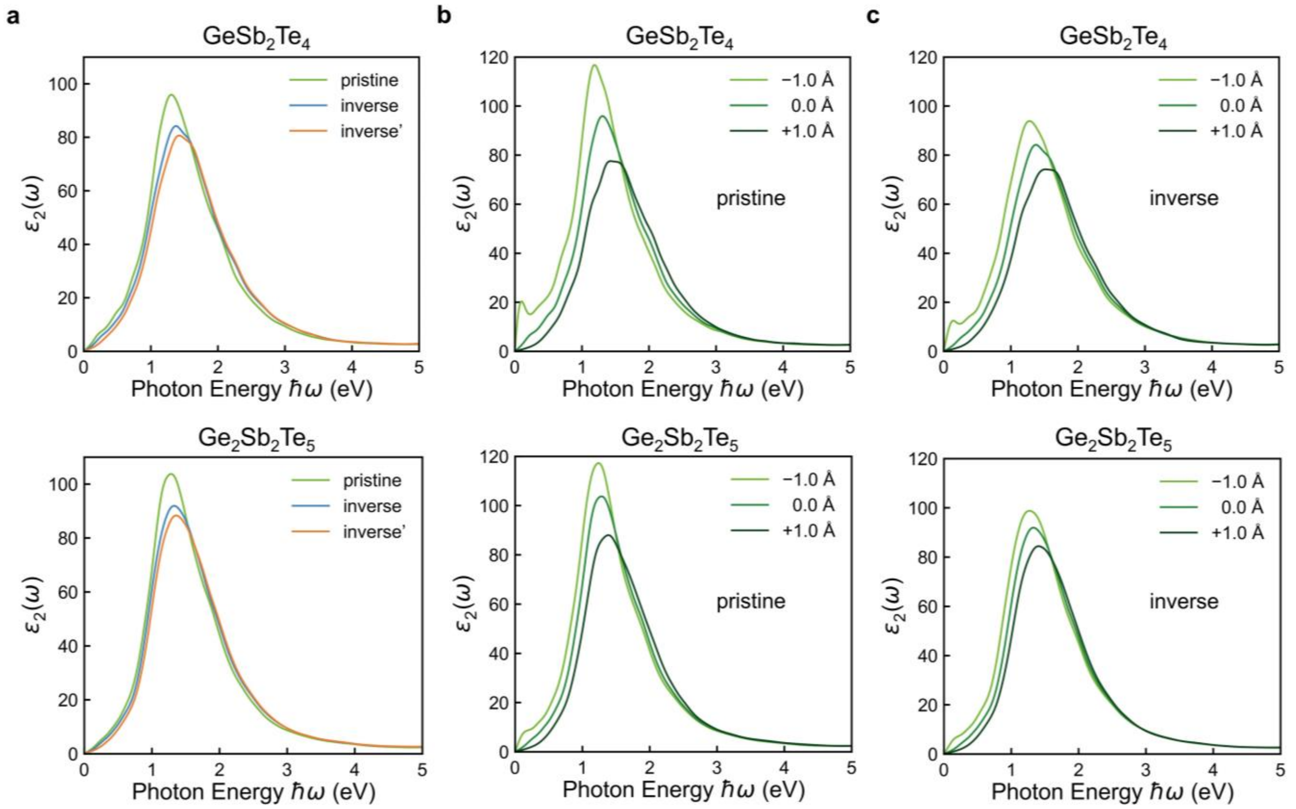

Figure S10. (a) The imaginary part (ε_2) of dielectric function of *t*-$GeSb_2Te_4$ and *t*-$Ge_2Sb_2Te_5$ with and without the inverse block. The corresponding atomic structures are shown in Figure S2. The changes in ε_2 under uniaxial strain for (b) the pristine structures and (c) the inverse structures.

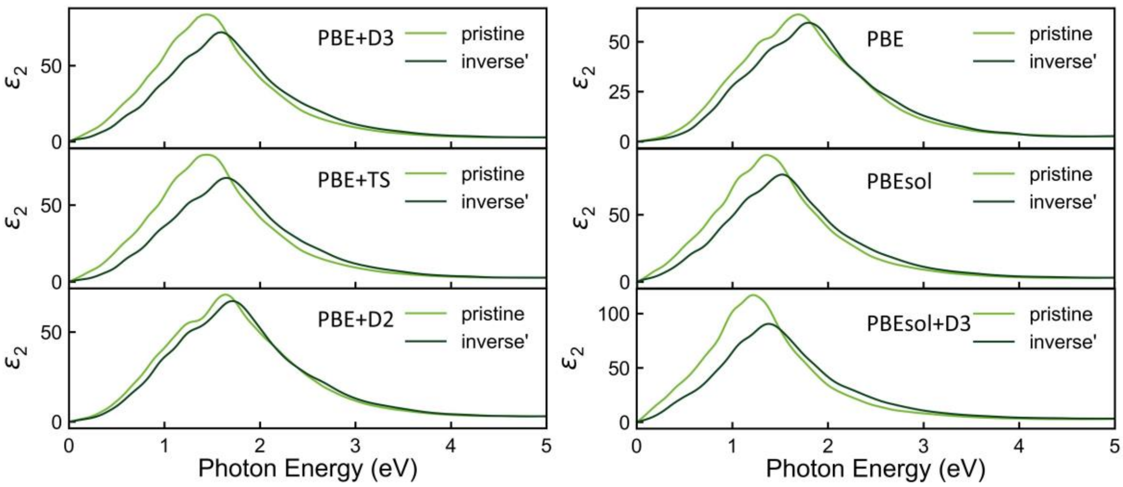

Figure S11. Calculations of $\varepsilon_2(\omega)$ using different combination of functional or vdW corrections.
